# People Welcomed This Innovation with Two Hands: A Qualitative Report of an mHealth Intervention for Community Case Management in Malawi

**DOI:** 10.5334/aogh.919

**Published:** 2019-04-25

**Authors:** Nicole Ide, Victoria Hardy, Griphin Chirambo, Ciara Heavin, Yvonne O’Connor, John O’Donoghue, Nikolaos Mastellos, Kanika Dharmayat, Bo Andersson, Sven Carlsson, Adamson Muula, Matthew Thompson

**Affiliations:** 1 University of Washington, US; 2 Mzuzu University, MW; 3 University College Cork, IE; 4 Imperial College London, GB; 5 Lund University, SE; 6 College of Medicine, MW

## Abstract

**Introduction:**

Community Case Management (CCM) aims to improve health outcomes among children under five with malaria, diarrhea, and pneumonia, but its effectiveness in Malawi is limited by inconsistent standards of delivery characteristic of paper-based interventions. This may lead to negative impacts on child health outcomes and inefficient use of health system resources. This study evaluated the acceptability and impact of the Supporting LIFE Community Case Management App (SL eCCM App) by Health Surveillance Assistants (HSAs) and caregivers in two districts of Northern Malawi.

**Methods:**

Data were collected through semi-structured interviews with HSAs and caregivers as part of a nested study within a larger trial. We used deductive and inductive approaches during data analysis. Relevant constructs were identified from the Consolidated Framework for Implementation Research and combined with emerging concepts from the data. The Framework Method was used to chart and explore data, leading to the development of themes.

**Results:**

Seventeen HSAs and 28 caregivers were interviewed. Participants were generally enthusiastic about the SL eCCM App. Nearly all HSAs expressed a preference for the App over routine paper-based CCM. Most HSAs claimed the App was more reliable and less error prone, facilitated more accurate diagnoses and treatment recommendations, and enhanced professional confidence and respect in the community. Some HSAs believed additional features would improve usability of the App, others identified mobile network or electricity shortages as barriers. Not all caregivers understood the purpose of the App, but most welcomed it as a health and technological advancement.

**Conclusion:**

The SL eCCM App is acceptable to both HSAs and caregivers, and in most cases, preferred, as it was believed to foster improvements in CCM delivery. Our findings suggest that mobile health interventions for CCM, such as the SL eCCM App, may have potential to improve the effectiveness and efficiency of care to children under five.

## Introduction

The mortality rate from preventable illnesses among children under five-years of age in Sub-Saharan Africa decreased by 46%, from 179 per 1,000 live births in 1990 to 96 in 2015 [1]. This decline has been particularly notable in Malawi where mortality rates fell by two thirds over this period, making Malawi one of the few countries to reach their target for Millennium Development Goal #4 [2,3,4]. Yet, Malawi continues to face disproportionate childhood morbidity and mortality, perpetuated by inequities in health service delivery [5].

The paper-based clinical decision aid, Community Case Management (CCM), was heralded as an intervention that could help low– and middle-income countries (LMICs) achieve equitable and effective healthcare [6]. However, deployment of CCM by community health workers (CHWs) has encountered bottlenecks to achieving desired patient management standards, including inadequate supervision, poor retention of CHWs, local barriers to care seeking by parents, limitations in drug supply chain, and poor data on utilization of services and outcomes from CCM [7,8]. These deficiencies in CCM delivery prevent potentially serious illnesses from being promptly recognized and managed, resulting in higher-level facilities (e.g. rural hospitals) being overwhelmed by sick children [9]. A functioning referral system for children unable to be managed by CHWs is vital for balancing resources and optimizing acute pediatric care. Therefore, strategies that enhance high quality CCM delivery in first-level community settings are a priority.

A growing body of evidence suggests that mobile health (mHealth) platforms for CCM could circumvent many of the limitations of current paper-based delivery. Previous studies investigating digitized versions of CCM demonstrated improved diagnostic accuracy and appropriateness of therapeutic decisions by CHWs compared to the paper based tool [10,11,12,13]. Additional benefits of digitized clinical decision rules over the paper based tools included: more thorough clinical assessments [14], improved communication to caregivers about management plans [15], increased caregiver confidence in CHWs’ competency [14], and integration of training videos and supervisory support [16]. However, the impact of interventions on CHWs’ referral patterns and caregivers’ subsequent health-seeking behavior, to our knowledge, has yet to be investigated. We sought to address this in a clinical trial [17]. We investigated the acceptability of delivering CCM via an mHealth solution among CHWs, known in Malawi as Health Surveillance Assistants (HSAs), and parents/caregivers (referred to as caregivers hereafter), with a particular emphasis on factors that might influence its impact on referrals from community clinics to higher-level health care facilities.

## Methods

### Design

This qualitative study was nested within a stepped-wedge cluster randomized trial comparing the Supporting LIFE (SL) electronic CCM application (SL eCCM App) plus paper CCM (intervention) [18], to paper CCM alone (control). We used a mixed deductive and inductive approach [19], using the Consolidated Framework for Implementation Research (CFIR) to guide the development of a matrix onto which excerpts were mapped through open coding. CFIR was selected as it provides a comprehensive approach for evaluating complex interventions and interpreting findings [20,21].

### Study Setting

The trial was conducted from October 2016 to February 2017 in Rumphi and Nkhata Bay districts, Northern Malawi (methods described in full elsewhere) [17]. Qualitative interviews were conducted with participants between January–February 2017 at village clinics, participants’ homes, or a community facility convenient to participants.

### Participant Population

Participants were HSAs operating an active village clinic within the targeted districts and caregivers (any accompanying adult aged 18 years and above) presenting to a participating village clinic with a sick child (aged ≥2 months to <5 years). All HSAs enrolled in the trial were eligible to be invited for interview. Only caregivers enrolled in the intervention phase were interviewed. As CCM was implemented in Malawi in 2008 [22], prior caregiver exposure to paper CCM was assumed.

### Intervention

Android smartphones were provided to all 102 participating HSAs. HSAs deployed paper-based CCM (control) followed by both the SL eCCM App plus paper CCM (intervention). Depending on the cluster to which HSAs were randomized, the duration of each phase was 2–7 weeks [17]. During the intervention, the App directed assessment and management, and the visit was documented in both the App and the village clinic register (VCR). Duplicate data entry procedures were necessary as monthly submission of physical records are mandated by the Malawian Ministry of Health (MoH).

### Sampling and Recruitment

Participants were informed prior to trial enrollment (at training workshops for HSAs and at presentation to village clinics for caregivers) that they might be contacted by the research team to participate in interviews. To ensure we explored a range of issues regarding use of the SL eCCM App in urban and more rural locations, 2–3 HSAs from each of the six clusters were purposively sampled by gender and geographical setting [23]. We randomly sampled between 4–5 caregivers from each cluster enrolled during the intervention phase by blindly hand selecting enrollment forms from their respective filing folder. The interviewer contacted HSAs and caregivers by phone or in person, explained his intent and involvement with the trial, and invited HSAs to participate in a semi-structured interview. To decrease potential for recall bias, we planned to conduct interviews within one week of trial completion for HSAs and two weeks from trial enrollment for caregivers. However, due to time and resource constraints, HSAs were interviewed within one month of completion, while all caregivers were contacted within two weeks. Our target sample size of 15 HSAs and 25 caregivers was determined *a priori* and informed by established research indicating that saturation typically occurs within 12 interviews [24]. There was scope to sample beyond this target if the research team believed themes had not been fully explored.

### Data Collection

A member of the study team fluent in the three common languages spoken by participants (Chichewa, Tumbuka, and Tonga) with prior interviewing experience conducted all interviews in participants’ preferred language. Using topic guides tailored for each participant group, interviews were conducted face-to-face and in private to provide space for open communication. Topic guides included open-ended questions and suggested prompts. Questions to HSAs involved their experience using the App compared to paper-based methods, barriers to using the App, trust in the App’s recommendations, and observed caregiver reactions. Questions to caregivers involved their experiences and attitudes towards the App, beliefs in the accuracy and of the App to correctly diagnose their child, and factors influencing caregiver compliance with referral advice. Interviews were audio-recorded with participant permission. Repeat interviews were not carried out, and field notes were not taken during interviews.

### Data Management

Audio files were translated into English by transcribers/translators fluent in the relevant languages. The transcribed text reflected the audio file as accurately as possible and included non-verbal utterances and suggestions for ambiguous sections in the original transcription. A study member fluent in the relevant languages checked each transcription against the original voice recording and translated texts for accuracy and removed identifiers. Transcripts were not returned to participants for comment or correction. Digital copies of the translated English text were uploaded into NVivo 11 to facilitate data analysis [25].

### Analytical Framework

Three authors participated in developing the analytical coding framework. The CFIR was used in conjunction with the authors’ existing knowledge acquired from a prior feasibility study [26], and the mHealth literature for CCM interventions in sub-Saharan Africa to help determine the key categories of our analytical framework *a priori*. We identified potentially applicable constructs (categories) within three CFIR domains: Intervention Characteristics, Inner Setting, and Characteristics of Individuals. These constructs were used to guide coding and were combined with additional categories which emerged from the data analysis (Table 1).

**Table 1:** Themes and Categories for Analysis.

THEME	Theme Definition	CATEGORY	Category Definition

**HSA Acceptability and Beliefs**	HSAs’ overall perceptions of the App and trust in the App to correctly direct clinical management	Evidence strength and quality*	HSAs’ perceptions of the quality and validity of evidence supporting beliefs that the App will produce desired outcomes
		Tension for change*	Degree to which participants perceive current paper CCM methods as intolerable or needing change
**Caregiver Acceptability and Beliefs**	Caregivers’ views on technology, their level of trust in the App, and any concerns about the intervention	Evidence strength and quality*	Caregivers’ perceptions of the quality and validity of evidence supporting beliefs that the App will produce desired outcomes
		Knowledge and beliefs about the App*	Caregivers’ attitudes toward and value placed on the App as well as familiarity with facts, truths, and principles related to the intervention
		**Mistrust, myths, and rumors**	Myths or rumors reported in the community regarding the SL project
**Technical and Clinical Characteristics of the App**	Features of the App that affect the service delivery process	**Impact on the conduct of clinical assessments**	App features that aid HSAs in their clinical assessment of patients
	(Only includes categories which are directly related to the App, not the phone or other aspects)	**Impact on the referral process**	Changes to the referral process due to the App
		**Learning curve**	Reports on HSAs’ perceptions of training and their ability to learn to use the App
		**Missing or desired features of the App**	Missing or desired features that make the App less desirable compared to standard care
		Relative advantage of the App over standard care*	Usability features of the App that are perceived as advantages over standard care
**Indirect Benefits and Challenges of the intervention**	Challenges and benefits reported as indirect results of the intervention (i.e. the technology and study procedures) (includes challenges not directly related to app)	**Challenges to sustained or continued use of the intervention**	Concerns or difficulties perceived to using the App on an ongoing basis, or challenges to its sustainability.
		Additional benefits from the intervention*	Perception of benefits or advantages from participating in the intervention, above and beyond any impacts on their clinical care provided.
		**Caregiver-HSA relations**	Any reported changes to the relationship between HSA and caregiver due to the intervention

* Categories taken from CFIR constructs. The names may have been slightly adapted.

### Data Analysis Method

We used the Framework Method to guide data analysis [27]. After transcription, three authors immersed themselves in the translated text, and a single author applied open coding to a small number of interviews [28]. A codebook defining these codes was created, and initial codes were tested for ‘fit’ against categories derived a priori from CFIR. Additional categories were developed to incorporate emerging (inductive) codes. A second author then read through the codebook and interviews, and after agreement of data interpretation, the remaining interviews were coded using the existing codebook, and any new codes were added as they emerged. After coding was complete, categories, codes, their definitions, and illustrative quotes were charted into a matrix and codes/categories were compared, contrasted, and organized repeatedly until major patterns (themes) became apparent. To further ensure the integrity of data interpretation, the final matrix was reviewed by the interviewer and feedback was incorporated. Participants were not contacted for further validation.

### Ethical Considerations

Informed consent was obtained from all HSAs (in writing) and caregivers (verbally) prior to participation in the trial. During consent, participants were provided with a full explanation of the trial, which included potential participation in this nested qualitative study and research team contact details. Participants were free to opt out of interviews at any time. The study was approved by the University of Washington (51750), Imperial College London (16IC3396), and the University of Malawi College of Medicine Research Ethics Committee (P.07/16/1984).

Reporting of this study adheres to the consolidated criteria for reporting qualitative research (COREQ) guidelines [29].

## Results

Overall, 45 in-depth interviews were conducted with: 17 HSAs (8 Nkhata Bay; 9 Rumphi) and 28 caregivers (14 in each district). Of these, two caregivers during interview claimed not to have observed the HSA using the App to guide assessment, and so we were unable to explore their experiences with the intervention. However, we still included data from these participants regarding their general impressions of the use of technology in their village clinic. No invited participants declined participation. Interview length averaged 23 minutes and ranged from 10–60 minutes.

We identified four main themes (see Table 1): 1) HSA Acceptability and Beliefs; 2) Caregiver Acceptability and Beliefs; 3) Technical and Clinical Characteristics of the App; and 4) Indirect Benefits and Challenges of the intervention.

## HSA Acceptability and Beliefs

We identified two categories for this theme based on HSAs’ overall perceptions of the App and trust in its ability to correctly direct clinical management.

### Evidence strength and quality

When asked if they trust the App’s clinical management recommendations, HSAs typically responded affirmatively. When asked to elaborate, they often explained that the results elicited were typically the same as when they used the VCR and that the App guided a more thorough examination of the child.

“Yes, it’s right, because whatever is contained in the phone is the same with what is in the register… The phone is more accurate because you are able to have a thorough examination of the child. You go step by step more carefully than with the register.” (HSA 10028)

However, a few mentioned that while they found the App’s recommendations to generally be correct, they did not blindly trust the results. Instead, they balanced the App’s recommendations with what they found using the VCR and/or prior knowledge.

“We do not only use advice from the phone, but also relate to what we learnt at school, and if they match, we know that it is the right thing to do.” (HSA 10005)

### Tension for change

Overall, HSAs were motivated to use the App. Several expressed enthusiasm and a desire to continue using it, rather than reverting back to paper-based CCM.

“People welcomed this innovation with two hands.” (HSA 10042)

“With the experience I have had during the (intervention), (using just the VCR) would mean going back to old ways which I don’t like.” (HSA 10005)

## Caregiver Acceptability and Beliefs

Within this second theme, we identified three categories based on caregivers’ overall acceptability and beliefs regarding the App.

### Evidence strength and quality

Caregiver reactions to the App’s validity was mixed but leaned towards favorable. In a few cases, caregivers believed that the standard of care received would not be influenced by the App.

“The treatment will be the same because, even before the phone, the (HSAs) were helping, and I know that, with the introduction of the phones, the help will be the same.” (Caregiver 32858)

Two caregivers expressed doubt regarding the ability to diagnose children using an App, which was attributed to unfamiliarity with the technology. For example:

“I still had trust but I also had doubts because it was my first time seeing him using a phone.” (Caregiver 34754)

Some HSAs noticed that caregivers were more easily convinced that their child required urgent referrals when using the App compared to the VCR only.

“(The App) has an impact because, when (caregivers) see you using a phone, they are quickly convinced that their child is really sick and needs attention.” (HSA 10014)

### Knowledge and beliefs about the App

Most caregivers thought the technology was a good development in the community and that it would help the HSAs in their work. Many welcomed this technology as the way of the future and felt it was acceptable in their community.

“I just thought it’s a new era… In the past, they were using transcription papers, now they have started using phones.” (Caregiver 35521)

A few caregivers believed HSAs were using the App to communicate with clinicians at higher-level health facilities regarding the treatment of their children. Others identified being able to telephone HSAs for advice when their child becomes acutely unwell as a main purpose of the technology.

“It might help in the future when my child is sick suddenly, I can call the (village clinic) in a haste.” (Caregiver 32103)

Caregivers did express concerns regarding the App being used in village clinics, such as concerns about the phone malfunctioning, HSAs losing focus on the patient and becoming too absorbed with the technical aspects of the technology, and unfamiliarity with the purpose of the App. Some did not understand that the phone was being used to support clinical assessment and decision-making; they assumed the HSA was “*playing around with it or doing his own business.” (HSA 35150)*

“When he was touching the phone, he was just touching it until the end when he finished everything.” (Caregiver 32103)

### Mistrust, myths, and rumors

Although uncommon, a small number of caregivers described feelings of mistrust in how their child’s information would be used:

“I didn’t know that when they enter the records into the phone, they are entering (my child) into the agency.” (Caregiver 35283)

“And I heard other people saying we should not give out our phone numbers to HSAs. They say such practices are connected to Satanism.” (Caregiver 30097)

## Technical and Clinical Characteristics of the App

We identified five categories related to the technical or clinical features of the SL eCCM App itself.

### Impact on the conduct of clinical assessments

Nearly all HSAs believed the App helped them conduct more accurate assessments by preventing mistakes, such as skipping questions or steps during the examination of the child, and with the breath counter feature.

“You can make a mistake on the paper sometimes. For example, on fast breathing, instead of marking on the under-one aged child you can mark it on the over-one; while with the phone, it gives a clearly marked range.” (HSA 10005)

In addition to preventing errors, the App reminded HSAs what to do after assessment of the child and provided automated treatment recommendations.

“The thing we like most is that at the end of treatment, the App reminds the user what to do, so when you are done you cannot forget what to do.” (HSA 10002)

Only one HSA and three caregivers mentioned the App delayed their care, but they did not elaborate on whether perceived delays were connected to operability of the App or due to the double data entry (i.e. App and VCR).

### Impact on the referral process

Overall, HSAs felt the App assisted their referral decisions. Some described cases where the App identified children who would not have been referred had the HSA not been using the App:

“In some cases, according to my own opinion, the child would not have been referred to a health center, but then I found that the phone decided the child should be referred.” (HSA 10062)

In other cases, the App identified children who could safely be treated at home without the need for referral:

“With the use of the phone, after entering data from the parent, it shows on the spot that the child should be referred and then we refer. Other times we may judge the condition to be critical when actually the child is fine and can be treated at home.” (HSA 10074)

For some HSAs the App provided a sense of support or back up for a referral decision:

“The phone would tell us that you can’t treat this case and that there is a need for referral. Then, you could just accept it and send the child as soon as possible.” (HSA 10043)

For others, it provided faster information than the VCR:

“(The App) could tell me that the child has danger sign. Thus, I could send the child as quickly as possible.” (HSA 10028)

Overall, nearly two thirds of HSAs believed the App made the referral process for a sick child easier (n = 9) and/or faster (n = 3). Another two HSAs felt the App facilitated more appropriate clinical referral decisions. The overall perceptions of the App’s impact on the number of children referred varied among HSAs. Some noted that the overall number of referrals increased while using the App, others believed the number decreased, and others perceived no change to the referral process.

### Learning curve

HSAs explained they valued the training provided by the research team, and while some had difficulties using the App initially, a period of a few hours appeared sufficient for most to feel adequately trained. They also explained having a few weeks to practice using the phone prior to the intervention was helpful.

“After the training, we tried to practice right away. During the first hour of practice, we had problems to operate. But two to three hours later we got used to it, and up to now, there is no problem at all.” (HSA 10062)

“They gave the phone to us to practice for three weeks and by the time of starting work we were already conversant with how to use it.” (HSA 10076)

Some HSAs described variability in the ability to learn how to use the App, noting they believed it was more difficult for older HSAs. As a result, they commented that some HSAs might need more time for training.

“(Our colleagues who are of older age) really take time to adapt. Now, as long as there is more training, during the training they will still adapt slowly at their own pace.” (HSA 10030)

“During the last training they really struggled to adapt, there is just a need to increase number of days for the training so that everyone should fully understand.” (HSA 10030)

### Missing or desired features of the App

Some HSAs identified features they wished had been included in the App. The most frequent comments involved medication dosing and access to data after syncing with the central database. Others included having a place to indicate drug stock outs, a field to enter malaria rapid diagnostic test (MRDT) results, and providing diagnosis/treatment recommendations for negative MRDT results.

“It cannot show the detailed prescription of each child, but only the type of medication needed…and it cannot even differentiate that this medication is for a child of a particular age.” (HSA 10073)

“If we synchronize data and we don’t remain with it, it all goes away. It could be a very good thing if it could be updated so that we should be able to revisit the data if the need arises.” (HSA 10014)

### Relative advantage of the App over standard care

When asked if they preferred using the App to the VCR, ten HSAs stated they would prefer to use the App in isolation, six preferred to use both the App and the VCR, and one HSA was inconclusive. A few usability features were cited by HSAs as benefits to using the App compared to paper-based CCM, including faster provision of care, portability, improved durability, and more efficient and easier monthly reporting to the District Health Officer (DHO).

“I was faster when assisting patients (with the App) than when I used the register only.” (HSA 10014)

“The phone is easy to carry, no matter how much data has been logged in, unlike registers. (The registers) are too heavy when we carry them in our bags.” (HSA 10032)

“It’s easy to send a report in time when using the phone.” (HSA 10005)

“The phone will go directly to the solution or to the problem that it has diagnosed, while a register will display everything, and then it is my duty to find out what exactly I am looking for” (HSA 10032).

## Indirect Benefits and Challenges of the intervention

This theme included three categories reporting common challenges and benefits experienced as a result of the intervention, related to the composite experience of the technology and study processes.

### Challenges to sustained or continued use of the intervention

The main challenge to the continued use of the SL eCCM App related to technological malfunctions and infrastructure-related limitations, such as mobile network coverage or electricity shortages in Malawi.

“When there is a lot of work, like writing, operating the phone, testing the child, sometimes the phone shuts down (freezes). That means starting all over.” (HSA 10019)

“The phones will turn off due to low battery and where we charge is very far.” (HSA 10073)

Related to this was the potential need for ongoing technical support, maintenance, and concern about who would provide this in the future if the research team was not doing this.

“A lot of things need assistance, and the government on its own cannot manage. For example, if the phone itself has problems, it means you cannot use the App.” (HSA 10014)

Some HSAs noted phone malfunctions occurred as a result of installing or using other applications, which could be a likely ongoing challenge.

“We put some applications in the phone not related to the work meant for it…and I have seen it as one of the problems which can make a phone to not operate properly in examining children.” (HSA 10073)

Finally, across all the sites taking part in the trial, two of the study phones were stolen during the intervention, although both were recovered after policeinvestigation.

### Additional benefits from the intervention

The introduction of smartphone technology was seen as a direct benefit to HSAs as it represented modern technology – the first such modern technology they had been provided in their village clinics.

“We are now equipped with this modern technology of which we didn’t know, but now we do.” (HSA 10062)

HSAs commented that using this technology increased their confidence, their level of respect, and feeling of professionalism in their communities.

“Most of the time, we HSAs sometimes were regarded as not very important, but with the introduction of the phone, we know that somewhere, somehow, we are also being considered as very important people in the society.” (HSA 10088)

### Caregiver-HSA relations

Some HSAs noted that the SL intervention seemed to prompt increased attendance in their clinics, but it was unclear if this was due to perceptions of an improved standard of care based on the App, or an improved relationship, or another reason.

“Most parents were happy because even though we were taking more time to examine their children, they could feel the job was done well.” (HSA 10030)

“Our relationship is improving, because I have noted that the turn up is good. Previously, they did not come in such large numbers.” (HSA 10014)

Other explanations included that this may have been driven by factors such as caregivers being incentivized to attend as they received soap as a participant in the trial, or simply out of curiosity about the new project being conducted at village clinics.

“Some came because they wanted to get soap as their friends did, and also to see how the child is examined through a phone.” (HSA 10076)

While eleven HSAs reported strengthened relationships with caregivers after they started using the App, three HSAs and most caregivers mentioned no change in the relationship. We did not identify reasons for these differing perceptions.

“Even nowadays with the use of cellphones, I don’t see any change in them.” (Caregiver 33296)

## Discussion

### Summary of findings

Overall, HSAs and caregivers found the SL eCCM App highly acceptable, and most HSAs were enthusiastic about using the App as a standalone method. According to HSAs, a key benefit of the App was improved reliability and reduced errors, as it prevented assessment items from being overlooked. This led them to believe the App encouraged more accurate clinical assessments. Additional benefits cited by HSAs included the portability of the device as well as heightened prestige associated with using technology in health care. Caregivers generally agreed that the care their child received either improved or remained the same under the App, and it did not have any negative impact on their level of trust or relationship with the HSA. Many caregivers expressed excitement in seeing technology being used in their village. Skepticism, where it existed among caregivers, was related to not understanding the rationale for the technology and concerns about HSAs becoming too focused on the App to the potential detriment of patient care. We were specifically interested in HSAs’ and caregivers’ perspectives on whether the intervention impacted the referral process. The majority of HSAs reported that the App increased their confidence in their referral decisions and simplified and/or quickened the process.

The main challenges associated with using the App included the omission of fields in the App present in the VCR (MRDT results, medication dosing) and the absence of additional features that would potentially enhance HSAs’ jobs (e.g. medication stock out reporting). However, external limitations were the most commonly cited challenges, such as poor network coverage hindering data synchronization, lack of access to a power supply to maintain battery life, and theft of devices which occurred twice throughout the course of the trial. A small number of HSAs and caregivers reported hearing misconceptions spread in the community about the project, although in most cases, it did not appear to have significant impacts on attendance to the village clinics or enrolment in the study.

Most caregivers did not cite barriers to receiving clinical advice based on the App. In fact, some HSAs remarked that attendance at their clinics increased during the intervention. However, the reasons for this increase cannot be determined from this study alone. Some HSAs offered explanations such as provision of research participant incentives (soap) or caregivers’ curiosity about the project; however, caregivers did not offer these explanations themselves during the interviews.

### Comparison with existing literature

Two qualitative studies from Tanzania evaluated user and caregiver perceptions of an electronic version of Integrated Management of Childhood Illness (IMCI), the tool that CCM is derived from [14,30]. Both reported similar findings to our study. Shao et al. (2015) found that health workers claimed the e-IMCI tool simplified their work, facilitated correct treatments, and maintained caregivers’ trust [30]. Mitchell et al. (2013) found that caregivers trusted the e-IMCI tool and that health workers preferred the tool over paper-based methods [12]. The health workers perceived the tool to be faster, simpler, and enabled them to follow the protocol more accurately. Our finding of HSAs’ perceived personal benefits, including greater confidence in treatment decisions, a higher sense of professionalism, and increased respect in the community were also identified as important motivating factors in previous studies [31].

In a review of challenges to health informatics in developing countries, Luna et al. (2014) describe similar challenges to those described by HSAs in our study [32], and a second review by Aranda-Jan (2014) noted similar infrastructure challenges to mHealth projects in Africa [33]. While the entire population of Malawi was reported in 2014 to be covered by a mobile-cellular network [34], only 32% are covered by at least a 3G mobile network [35], and only 11.0% of the population (or 4.0% of rural populations) have regular access to electricity [36]. In order to maximize the sustainability and success of future mHealth interventions, these challenges must be addressed, for example, through the use of solar panels or enabling data entry/storage without a network connection [37].

Though very infrequent, there were a few reported misconceptions and hints of mistrust in the community about the Supporting LIFE project. Concerns regarding the recording of patient data in phone suggest that some parents did not realize the VCR was already used to record and report this same data. Mistrust may also have stemmed from misinformation in the community regarding mobile phones and health IT generally. Further, the misconceptions noted may reflect wider concerns and suspicions about health research. These perceptions in the community may have consequences in a trial community, and potentially for the continued sustainability of an intervention [38]. From its inception, the SL project engaged extensively with local researchers and NGOs as part of the study to facilitate communication and reduce misconceptions wherever possible.

### Strengths and Limitations

A key strength is the interdisciplinary nature of the study, including a research team with expertise from sociological, health care, and information technology perspectives, and interviews from the perspectives of both caregivers and CHWs. These ranges of perspective are often lacking in mHealth projects conducted in LMICs [39]. In addition, we sampled participants from varied geographic settings within the region of rural northern Malawi, and HSAs and parents with varied prior knowledge of smartphone technology. These factors enhance the transferability of our results within Malawi and similar settings. Selecting a Malawian interviewer who understands the culture and languages and who was also familiar with the wider context of the study was important for encouraging participants to reflect and express their views candidly. However, because he is a well-respected clinician and academic locally, this may have reduced participants’ willingness to disclose negative opinions.

A limitation of the study is the short duration of the trial. HSAs had only a limited time using the App (2–7 weeks) during which time most caregivers only interfaced with the HSA (and App) once. We are therefore unable to determine if prolonged use of the App would alter perceptions and experiences among HSAs and caregivers. Nevertheless, the study is one of the longest clinical trials of an mHealth intervention to date in Malawi. A further limitation is that we were unable to evaluate the use of the App without need for the HSA to duplicate their clinical assessment using the paper register, as the latter was required for reporting to the Malawian Ministry of Health. Finally, although sampling methods ensured a representative sample was taken, participant demographic characteristics were not recorded at the time of interview.

### Implications of our findings

The findings of this study provide new evidence to support decisions on implementation of mHealth programs in Malawi. The lessons learned will be valuable to health providers, policy makers, as well as for future research. The ease with which HSAs adopted eCCM and its acceptability to both HSAs and caregivers suggests that the intervention could be used instead of the village clinic paper register. The App also has potential to improve the efficiency of disease surveillance data and clinical activity reporting, which could lead to faster outbreak reporting and better coordination of drug supply.

Although the SL trial was comparatively large in scale and duration, a number of research questions remain. Implementation research of a wide roll out of the App and any changes over time in practice and clinical care need to be evaluated. Over the long term, sustainability, cost-effectiveness, unanticipated consequences and positive outcomes, and variation by geographical region also need to be considered. Additional research questions need to address the integration of the SL eCCM App with HSA’s other community responsibilities and ensuring data compatibility with the national health data system.

## Conclusion

The perceptions and experiences of stakeholders within the local environment can be just as consequential as the clinical capabilities of the SL eCCM App. A supportive community, buy-in from HSAs and caregivers, and minimal usability challenges will be essential for the future success of this application. This study suggests that HSAs and caregivers are ready and willing to adopt mHealth applications as a decision support tool; however, sustainable solutions more compatible with end-user needs and existing infrastructure still must be addressed to ensure its long-term success.
